# Risk of coronary stenosis after adjuvant radiotherapy for breast cancer

**DOI:** 10.1007/s00066-022-01927-0

**Published:** 2022-04-07

**Authors:** A.-K. Wennstig, H. Garmo, L. Wadsten, B. Lagerqvist, I. Fredriksson, L. Holmberg, C. Blomqvist, G. Nilsson, M. Sund

**Affiliations:** 1 Department of Surgical and Perioperative Sciences/Surgery, Umeå University, Umeå, Sweden; 2 Department of Oncology, Sundsvall Hospital, Sundsvall, Sweden; 3 Regional Cancer Center, Uppsala University/Uppsala University Hospital, Uppsala, Sweden; 4 Department of Surgery, Sundsvall Hospital, Sundsvall, Sweden; 5 Department of Medical Sciences, Uppsala University, Uppsala, Sweden; 6 Department of Molecular Medicine and Surgery, Karolinska Institutet, Stockholm, Sweden; 7 Department of Breast‑, Endocrine tumors and Sarcoma, Karolinska University Hospital, Stockholm, Sweden; 8 Department of Surgical Sciences, Uppsala University, Uppsala, Sweden; 9 Translational Oncology & Urology Research (TOUR), School of Cancer and Pharmaceutical Sciences, King’s College London, London, UK; 10 Department of Oncology, Örebro University, University Hospital, Örebro, Sweden; 11 Department of Immunology, Genetics and Pathology, Section of Experimental and Clinical Oncology, Uppsala University, University Hospital, Uppsala, Sweden; 12 Department of Oncology, Gävle Hospital, Gävle, Sweden; 13 Department of Oncology, Visby Hospital, Visby, Sweden; 14 Department of Surgery, University of Helsinki and Helsinki University Hospital, Helsinki, Finland

**Keywords:** Side effects, Survivorship, Ischemic heart disease, LAD (left anterior descending artery), PCI (percutaneous coronary intervention)

## Abstract

**Purpose:**

Adjuvant radiotherapy (RT) for breast cancer is associated with an increased risk of ischemic heart disease. We examined the risk of coronary artery stenosis in a large cohort of women with breast cancer receiving adjuvant RT.

**Methods:**

A cohort of women diagnosed with breast cancer between 1992 and 2012 in three Swedish health care regions (*n* = 57,066) were linked to the Swedish Coronary Angiography and Angioplasty Registry (SCAAR) to identify women receiving RT who subsequently underwent a percutaneous coronary intervention (PCI) due to coronary stenosis. Cox regression analyses were performed to examine risk of a coronary intervention and competing risk analyses were performed to calculate cumulative incidence.

**Results:**

A total of 649 women with left-sided breast cancer and 494 women with right-sided breast cancer underwent a PCI. Women who received left-sided RT had a significantly higher risk of a PCI in the left anterior descending artery (LAD) compared to women who received right-sided RT, hazard ratio (HR) 1.44 (95% confidence interval [CI] 1.21–1.77, *p* < 0.001). For the proximal, mid, and distal LAD, the HRs were 1.60 (95% CI 1.22–2.10), 1.38 (95% CI 1.07–1.78), and 2.43 (95% CI 1.33–4.41), respectively. The cumulative incidence of coronary events at 25 years from breast cancer diagnosis were 7.0% in women receiving left-sided RT and 4.4% in women receiving right-sided RT.

**Conclusion:**

Implementing and further developing techniques that lower cardiac doses is important in order to reduce the risk of long-term side effects of adjuvant RT for breast cancer.

**Supplementary Information:**

The online version of this article (10.1007/s00066-022-01927-0) contains supplementary material, which is available to authorized users.

## Introduction

There is accumulating evidence that radiotherapy (RT) for breast cancer (BC) can lead to subsequent ischemic heart disease (IHD) [1–4]. The incidence of BC is increasing but prognosis has improved substantially due to earlier detection by screening mammography and to more effective adjuvant therapies, including RT [2, 5, 6]. As more patients become survivors, the balance between benefit and harm, including potential long-term side effects, of BC treatments is important.

The incidental cardiac radiation doses are generally higher in left-sided RT compared to right-sided RT, and several studies show an increased risk of IHD after RT in women with left-sided compared to right-sided BC [1, 7, 8]. However, studies in more recently treated patients show conflicting results, and since RT-induced IHD is generally considered to be a late event, the follow-up may be too short draw firm conclusions [9–12]. A higher incidence of coronary stenosis in the left anterior descending artery (LAD) has been reported after RT of left-sided compared to right-sided BC, and some studies show a relationship between LAD radiation dose and the risk of developing coronary stenosis [13–17]. Increasing awareness of long-term cardiac toxicity and the development of new radiation techniques have contributed to reduced cardiac radiation dose exposure over recent decades. The anterior part of the heart, including the LAD, may still receive considerable doses in left-sided RT [15, 18–23].

Retrospective studies often rely on the validity of diagnosis coding in patient charts and in population-based registers. Since symptoms from the chest are common and multifactorial, there is a risk that misdiagnosis or overdiagnosis can influence the results. In this study, we examined the risk of coronary stenosis in women who received adjuvant RT for BC, and who were subsequently referred to coronary angiography and percutaneous coronary intervention (PCI).

## Methods

### Study population

The study population was created by selecting all women diagnosed with BC between 1992 and 2012 in three of Sweden’s six health care regions: Uppsala-Örebro, Stockholm, and the northern region. Since the 1970s, regional breast cancer registries have collected information regarding histopathological data and BC treatments in Swedish women with BC, and from 2008, all BC patients have been registered in the National Quality Registry for Breast Cancer [24]. Data on women with BC were then linked to the Swedish Coronary Angiography and Angioplasty Registry (SCAAR) to identify women with BC who subsequently underwent coronary angiography. The SCAAR is a part of the nationwide Swedish cardiac register SWEDEHEART and contains information on all patients who are referred to angiography and angioplasty. The register comprises information on baseline characteristics and detailed descriptions of angiographic findings and coronary interventions [25].

Women with a pathological coronary finding prior to or within 180 days from BC diagnosis based on registrations in the SCAAR or other SWEDEHEART registers were excluded. Women who had undergone coronary angiography with normal findings or only atheromatosis prior to or within 180 days from BC diagnosis were, however, included. Women with bilateral BC, with unknown BC laterality, with previously irradiated in situ BC, and with metastatic disease at BC diagnosis were excluded. A flowchart for study inclusion is shown in Figure S‑1.

### Statistical methods

Two types of time-to-event analyses were performed: time to a coronary finding and time to a PCI. Date of inclusion was defined as 180 days from BC diagnosis, and the women were followed to the date of first coronary finding/PCI in the SCAAR, death, migration, or end of follow-up (15 January 2021), whichever came first. Women with a normal coronary finding in SCAAR at the start of follow-up were excluded from the time-to-coronary-finding analysis.

To assess the cumulative incidence of coronary findings, competing risk analyses were performed censoring for death and end of follow-up, and considering the various types of coronary findings as competing risks. Findings were classified as follows: left main coronary artery (LMCA) disease, one-vessel disease (LMCA excluded), two-vessel disease (LMCA excluded), three-vessel disease (LMCA excluded), coronary atheromatosis, or normal angiographic findings. Results were presented using stacked cumulative incidences of findings presented separately for women diagnosed with right- and left-sided BC. The analyses were performed for the whole cohort and stratified by RT.

To estimate the risk of a PCI, Cox proportional hazard regression analysis was performed by comparing women who had received left-sided RT to women who had undergone right-sided RT. The analyses were performed separately for the LMCA, the LAD (proximal, mid, and distal LAD), the right coronary artery (RCA) (proximal, mid, and distal RCA), and the left circumflex artery (LCx). The proximal, mid, and distal LAD, and proximal, mid, and distal RCA were also analyzed separately. Coronary artery segments not included in the LAD, RCA, or LCx were grouped together and defined as “other locations.” The analyses were stratified for RT, type of surgery, and lymph node status recorded in the BC registries. Analyses were also performed separately on the risk of a PCI in the LAD in women treated between 1992 and 2001, and in women treated between 2002 and 2012. Separate analyses on the risk of a PCI in the RCA, the LCx, or other location were performed for women receiving RT, stratified for the presence or absence of a simultaneous PCI in the LAD.

The risk of a PCI in the LAD and RCA, comparing women receiving RT to women not receiving RT for right-sided BC and left-sided BC, respectively, was also assessed by Cox regression analysis. These analyses were adjusted for type of surgery, chemotherapy, and endocrine therapy, and age was used as the timescale.

All analyses were performed using the statistical software R [26].

## Results

### Study population

The study population consisted of 57,066 women with BC; 27,645 with right-sided BC and 29,421 with left-sided BC (Table 1). The median age at BC diagnosis was 61 years. No major differences were seen in tumor characteristics or cancer treatments between right- and left-sided BC. The majority of the women were diagnosed with T1 or T2 tumors (47.2 and 29.6%, respectively). Of those with data on lymph node involvement, 57.0% had lymph node-negative disease (N0) and 32.3% had lymph node-positive disease (N1-3, and N4+). Radiotherapy was given in 63.5% of the women overall, with 45.0% of the women treated with breast-conserving surgery and subsequent RT, and 17.8% with mastectomy and RT. Women receiving RT were younger and followed for 25 years from BC diagnosis. Women not receiving RT were older and, due to deaths, only followed for 20 years from BC diagnosis.

**Table 1:** Characteristics of the study population

	Right BC (*n* = 27,645)		Left BC (*n* = 29,421)		Total (*n* = 57,066)	
*Age median, (Q*_*1*_*–Q*_*3*_*) years*	61	(52–71)	61	(52–71)	61	(52–71)
*T‑stage, n (%)*						
T0	4134	(15.0)	4299	(14.6)	8433	(14.8)
T1	13,111	(47.4)	13,825	(47.0)	26,936	(47.2)
T2	8124	(29.4)	8794	(29.9)	16,918	(29.6)
T3	1233	(4.5)	1343	(4.6)	2576	(4.5)
T4	483	(1.7)	517	(1.8)	1000	(1.8)
TX	560	(2.0)	643	(2.2)	1203	(2.1)
*Previous angiography, n (%)*						
No angiography	27,440	(99.3)	29,220	(99.3)	56,660	(99.3)
Normal angiography	205	(0.7)	201	(0.7)	406	(0.7)
*N‑stage, n (%)*						
N0	15,843	(57.3)	16,693	(56.7)	32,536	(57.0)
N1–3	5916	(21.4)	6300	(21.4)	12,216	(21.4)
N4+	2966	(10.7)	3252	(11.1)	6218	(10.9)
NX	2920	(10.6)	3176	(10.8)	6096	(10.7)
*ER status, n (%)*						
ER+	18,813	(68.1)	19,633	(66.7)	38,446	(67.4)
ER−	3873	(14.0)	4415	(15.0)	8288	(14.5)
Missing	4959	(17.9)	5373	(18.3)	10,332	(18.1)
*PR status, n (%)*						
PR+	15,626	(56.5)	16,263	(55.3)	31,889	(55.9)
PR−	6767	(24.5)	7470	(25.4)	14,237	(24.9)
Missing	5252	(19.0)	5688	(19.3)	10,940	(19.2)
*Surgery, n (%)*						
No surgery	865	(3.1)	942	(3.2)	1807	(3.2)
BCS	15,527	(56.2)	16,066	(54.6)	31,593	(55.4)
Mastectomy	10,691	(38.7)	11,805	(40.1)	22,496	(39.4)
Missing	562	(2.0)	608	(2.1)	1170	(2.1)
*Radiotherapy, n (%)*						
Yes	17,663	(63.9)	18,568	(63.1)	36,231	(63.5)
*Surgery/RT, n (%)*						
BCS + RT	12,652	(45.8)	13,012	(44.2)	25,664	(45.0)
Mastectomy + RT	4827	(17.5)	5358	(18.2)	10,185	(17.8)
BCS no RT	2875	(10.4)	3054	(10.4)	5929	(10.4)
Mastectomy no RT	5864	(21.2)	6447	(21.9)	12,311	(21.6)
Other combinations	1427	(5.2)	1550	(5.3)	2977	(5.2)
*Endocrine therapy, n (%)*						
Yes	17,195	(62.2)	18,088	(61.5)	35,283	(61.8)
Chemotherapy, *n* (%)						
Yes	7805	(28.2)	8343	(28.4)	16,148	(28.3)
*Trastuzumab, n (%)*^*a*^						
Yes	897	(7.6)	1009	(8.0)	1906	(7.8)

Baseline characteristics for women with breast cancer (BC) stratified by laterality of BC

*T stage* tumor size, *N status* pathological lymph nodes, *ER status* estrogen receptor status, *PR status* progesterone receptor status, *Q* quartile, *BCS* breast conserving surgery, *RT* radiotherapy

^a^Data only available for women diagnosed 2005–2012

A total of 1572 women with left-sided BC and 1322 women with right-sided BC had a registration in the SCAAR. The median follow-up time from BC diagnosis to registration in SCAAR was 11.6 years (Q1 = 7.5 years, Q3 = 17.1 years), and 1% of the women had a registration in SCAAR after more than 27 years. One-vessel disease was the most common pathologic angiographic finding. A pathologic angiography finding was found in 767 women with left-sided BC and in 606 women with right-sided BC (Table S-1). Out of these patients, 649 women with left-sided BC and 494 women with right-sided BC underwent a PCI (data not shown).

### Cumulative incidence of coronary findings

**Fig. 1:**
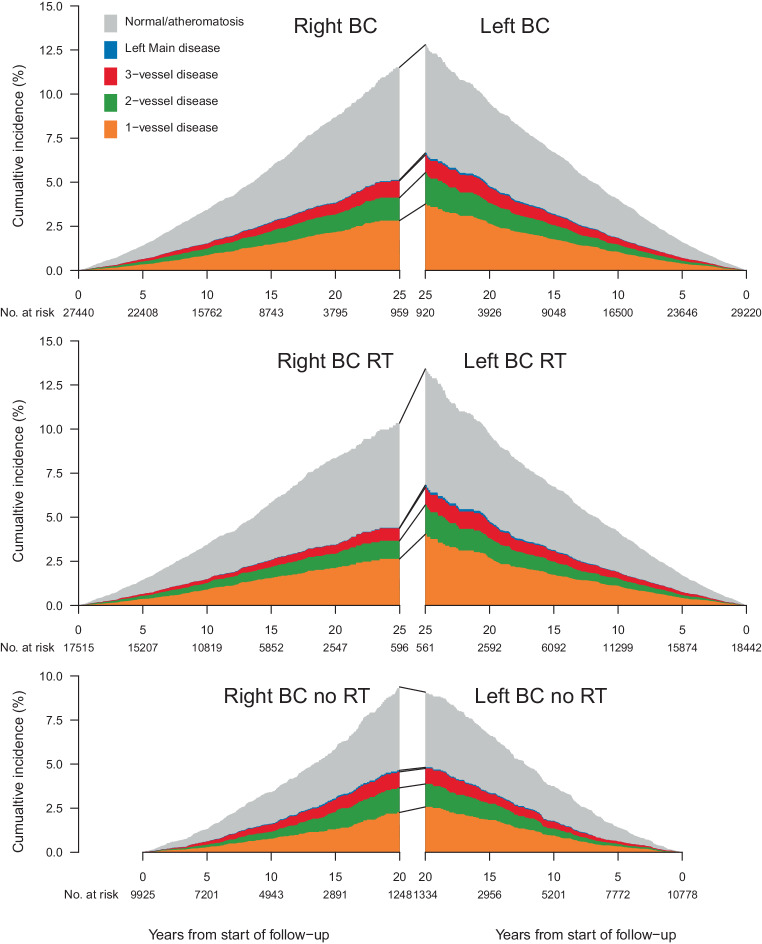
Cumulative incidence of coronary findings in women with breast cancer (*BC*), stratified by BC laterality and radiotherapy (*RT*). *Left Main* left main coronary artery

The cumulative incidence of a first coronary finding for right-sided and left-sided BC stratified by RT, visualizing the distribution of coronary findings, is shown in Fig. 1.

Women receiving left-sided RT had a higher cumulative incidence of coronary findings compared to women receiving right-sided RT, and the risk increased with longer follow-up. The cumulative incidence for a pathologic coronary finding, regardless of the extent of disease, at 10 years from BC diagnosis was 1.9% in women receiving left-sided RT, and 1.5% in women receiving right-sided RT. At 25 years from BC diagnosis, the cumulative incidence for a pathologic coronary finding was 7.0% in women receiving left-sided RT compared to 4.4% for women receiving right-sided RT. When divided into different extents of lymph node involvement, the cumulative incidence for a pathologic coronary finding at 10 years from BC diagnosis for N0, N 1–3, and N 4+ was 1.9%, 1.7%, and 2.0%, respectively, in women receiving left-sided RT, and 1.4%, 1.6%, and 1.7% in women receiving right-sided RT. At 25 years from BC diagnosis, the cumulative incidence for a pathologic coronary finding was 6.4%, 6.9%, and 9.9% for women receiving left-sided RT, and 4.5%, 4.6%, and 4.0% for women receiving right-sided RT, respectively (data not shown). In women not receiving RT, no major differences in the cumulative incidence of coronary events were seen (Fig. 1).

### Risk of percutaneous coronary intervention

**Fig. 2:**
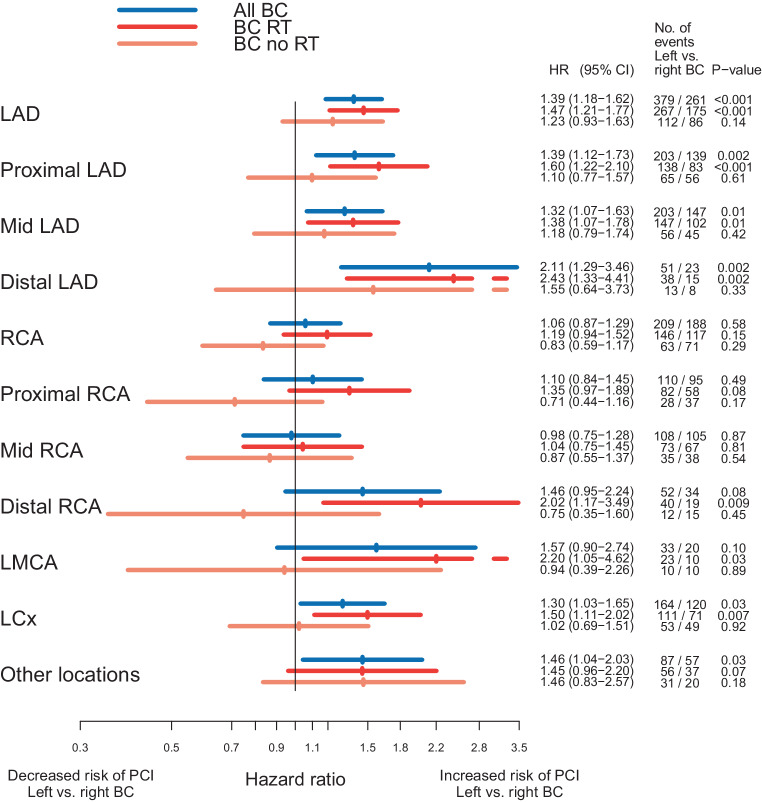
Risk of having a percutaneous coronary intervention (*PCI*) stratified by radiotherapy in women with breast cancer (*BC*) receiving left-sided radiotherapy (*RT*) compared to women with BC receiving right-sided RT. *LAD* left anterior descending artery, *RCA* right coronary artery, *LMCA* left main coronary artery, *LCx* left circumflex artery, *HR* hazard ratio, *CI* confidence interval

**Fig. 3:**
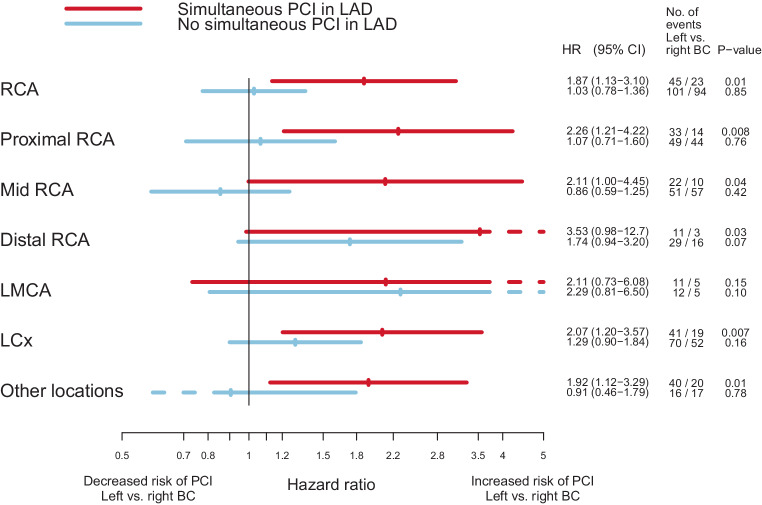
Risk of a percutaneous coronary intervention (*PCI*) stratified by a simultaneous intervention in the left anterior descending artery (*LAD*) in women with breast cancer (*BC*) receiving left-sided radiotherapy (*RT*) compared to women with BC receiving right-sided RT. *RCA* right coronary artery, *LMCA* left main coronary artery, *LCx* left circumflex artery, *HR* hazard ratio, *CI* confidence interval

The risk of a PCI in the different coronary arteries and segments, comparing left-sided to right-sided BC and stratified by RT is shown in Fig. 2. In women receiving RT, the Hazard ratios (HRs) for a PCI were 1.47 (95% confidence interval [CI] 1.21–1.77) for the whole LAD, and when divided into segments, 1.60 (95% CI 1.22–2.10), 1.38 (95% CI 1.07–1.78), and 2.43 (95% CI 1.33–4.41) for the proximal, mid, and distal LAD after left-sided versus right-sided RT, respectively. When stratified on year of BC diagnosis, the HR of a PCI in the LAD was 1.66 (95% CI 1.25–2.20) in women receiving RT between 1992 and 2001 and 1.31 (95% CI 1.01–1.70) in women receiving RT between 2002 and 2012, in left-sided RT compared to right-sided RT (data not shown). Increased HRs of a PCI in women receiving left-sided RT were also seen in the distal RCA and the LCx, 2.02 (95% CI 1.17–3.49) and 1.50 (95% CI 1.11–2.02), respectively. When the analyses were performed stratified by a simultaneous PCI in the LAD, no significant increase in HRs for these coronary arteries were seen in women with left-sided BC compared to right-sided BC (Fig. 3). The risk of a PCI comparing women receiving left- to right-sided RT stratified by pathologic lymph node stage is shown in Figure S‑2. In the LAD, the HRs for a PCI increased with more advanced nodal disease. The HRs for left-sided BC compared to right-sided RT were 1.32 (95% CI 1.03–1.69), 1.54 (95% CI 1.03–2.32), and 1.91 (95% CI 1.07–3.40) for N0, N1–3, and N4+, respectively. No major difference in the risk of a PCI between different types of surgery was seen (Figure S-3).

When comparing women receiving RT to women not receiving RT, the adjusted HR for a PCI was 1.29 (95% CI 1.01–1.65) for the LAD and 1.25 (95% CI 0.90–1.73) for the RCA in women with left-sided BC. In women with right-sided BC, the adjusted HR was 1.07 (95% CI 0.81–1.43) for the LAD and 0.82 (95% CI 0.59–1.14) for the RCA (Table 2).

**Table 2:** Risk of having a percutaneous coronary intervention in women with breast cancer receiving radiotherapy compared to women not receiving radiotherapy

	Right sided BC RT vs. no RT		Left sided BC RT vs. no RT	
	HR (95% CI) Crude	HR (95% CI) Adjusted^a^	HR (95%CI) Crude	HR (95% CI) Adjusted^a^
LAD	1.07 (0.82–1.39)	1.07 (0.81–1.43)	1.31 (1.05–1.65)	1.29 (1.01–1.65)
RCA	0.82 (0.61–1.11)	0.82 (0.59–1.14)	1.24 (0.92–1.67)	1.25 (0.90–1.73)

*BC* breast cancer, *RT* radiotherapy, *vs.* versus, *HR* hazard ratio, *CI* confidence interval, *LAD* left anterior descending artery, *RCA* right coronary artery

^a^Adjusted for type of surgery (mastectomy/breast-conserving surgery/no surgery), chemotherapy (yes/no), and endocrine therapy (yes/no)

## Discussion

The main finding of the present study was an increased risk of a PCI in the LAD in women receiving left-sided RT compared to right-sided RT for BC (HR of 1.47, 95% CI 1.21–1.77). The risk of a PCI increased with more advanced nodal disease and was most pronounced in the distal LAD (HR 2.43, 95% CI 1.33–4.41). An increase in the cumulative incidence of coronary findings in women receiving left-sided RT compared to right-sided RT was apparent at 10 years from BC diagnosis and increased with longer follow-up, with an absolute increase in risk of 0.4% at 10 years of follow-up, and of 2.6% at 25 years of follow-up.

To our knowledge, this is the largest study to evaluate the risk of coronary findings and PCI in patients who undergo coronary angiography after previous adjuvant RT for BC. The registrations in the SCAAR give information on the exact localization of a PCI, and also indicate that these findings were of clinical significance to the patients.

Many previous studies on radiation-induced cardiac disease rely on diagnoses coded in patient charts and in population-based registers, and the accuracy of these registrations may affect the validity of the results [1, 7, 27]. Diagnosing IHD and heart failure can be challenging in clinical practice, as symptoms associated with cardiac diseases are common and multifactorial, and the risk of both over- and misdiagnosis must be considered [28, 29]. A strength with the present study is that all coronary findings and interventions were confirmed by coronary angiography and classified by cardiologists in the SCAAR. The register has been previously validated, with high coverage for patients undergoing coronary angiography and PCI [25].

Several weaknesses of the study need to be addressed. Information on individual radiation doses and fields were not available. However, most women likely received a dose of 2 Gray (Gy) to 50 Gy over 5 weeks, according to regional and national BC treatment guidelines throughout most of the study period [30]. Most women with axillary lymph node metastases also received RT to the axilla, the supraclavicular fossa, and in some cases to the internal mammary nodes (IMN). Deep inspiration breath hold (DIBH) techniques are shown to lower the radiation doses to heart and LAD [20, 21] and the risk of coronary events may be lower if DIBH techniques are used. The women in the present study were treated before these techniques were implemented in Sweden. The comparison of left-sided RT to right-sided RT is widely acknowledged in studies on cardiac side effects of RT [1, 7, 11]. These risk estimates may, however, underestimate the total risk of coronary disease, since cardiac doses after right-sided RT can also be considerable, especially if the IMN are included in the radiation target [20].

There are some recently published studies reporting coronary events after RT, registered by coronary angiography or coronary computed tomography angiography (CCTA) [9, 15, 17]. In a study by Tagami et al. [17], 94 patients treated with RT and subsequently diagnosed with coronary stenosis on CCTA were identified. In women treated with left-sided RT, a statistically significantly increased risk of coronary disease was seen in the LAD and the mean LAD radiation dose correlated with the risk of coronary disease. Based on these results, the authors suggested that ∼3 Gy may be a reasonable constraint for LAD doses at RT planning [17]. Since individual radiation doses were not available in the present study, we could not estimate risk of coronary stenosis per Gy. On the other hand, the present study consisted of a considerably larger cohort of women than that of Tagami et al. [17]. In a previous study by our group based partly on the same BC cohort as the present study, radiation dosimetry was studied in 182 women diagnosed with a coronary stenosis subsequent to RT. For these women, the mean median dose to the LAD was 10.9 Gy in left-sided RT [15]. In the study by Tagami et al., women receiving left-sided RT had a higher incidence of coronary findings in RCA compared to women receiving right-sided RT [17]. Individual differences in anatomy were discussed as one explanation for this finding, or the abscopal effect on tissues beyond the area of treatment. The analyses in the present study show a similar pattern, with an increased risk of PCI in the RCA in left-sided RT. However, when the analyses were stratified for a simultaneous PCI in the LAD, this increase in risk was no longer evident. These findings may be due to less clinically relevant coronary stenoses in the RCA that are detected and treated as a result of a symptomatic stenosis in the LAD.

In a study by Milo et al., a large cohort of Danish women treated with RT for BC between 1999 and 2016 was studied [9]. An increased incidence rate ratio for a cardiac event in left- versus right-sided RT of 1.44 (95% CI 1.8–2.4) was seen in women treated before computed tomography (CT)-based RT planning was introduced in Denmark (1999–2007), findings that are in line with the results of the present study. In women treated with CT-based RT between 2008 and 2016, however, no increased risk of cardiac events was observed in left-sided vs right-sided RT. In contrast to the study by Milo et al., we could also show the specific location of the coronary stenosis, and by this show an even higher risk of PCI in parts of the LAD, which is a strength of the present study.

The results of this study stress the importance of implementing and further developing RT techniques that lower the cardiac doses to reduce the risk of long-term side effects that impair health and quality of life for BC survivors.

## Supplementary Information

The supplementary information section include: Flow chart showing the selection of the study population (Figure S-1), Distribution of angiography findings (Table S-1), Risk of having a percutaneous coronary intervention stratified by pathological lymph node stage (Figure S-2), and Risk of having a percutaneous coronary intervention stratified by type of surgery (Figure S-3).
