# Supplementary material for: Risk of coronary stenosis after adjuvant radiotherapy for breast cancer
Source: Strahlenther Onkol. 2022 Apr 7;198(7):630–8. doi: 10.1007/s00066-022-01927-0 (PMC9217856; doi:10.1007/s00066-022-01927-0)
Supplement: Supplementary file 1 — The supplementary information section include: Flow chart showing the selection of the study population (Figure S-1), Distribution of angiography findings (Table S-1), Risk of having a percutaneous coronary intervention stratified by pathological lymph node stage (Figure S-2), and Risk of having a percutaneous coronary intervention stratified by type of surgery (Figure S-3). [file 66_2022_1927_MOESM1_ESM.docx]

**Supplementary material**

**Figure S-1**

**Flow chart showing the selection of the study population**

*Breast cancer (BC), number (n), radiotherapy (RT), coronary artery by-pass grafting (CABG), percutaneous coronary intervention (PCI), myocardial infarction (MI).*

*# According to SCAAR or SWEDEHEART.*

**Table S-1**

**Distribution of angiography findings**

|  | Right-sided BC no RT | Left-sided BC  no RT | Right-sided BC RT | Left-sided BC  RT |
| --- | --- | --- | --- | --- |
| 1-vessel disease | 107 | 133 | 224 | 295 |
| 2-vessel disease | 65 | 69 | 93 | 117 |
| 3-vessel disease | 53 | 47 | 56 | 92 |
| LMCA disease | 5 | 4 | 3 | 10 |
| Normal/only atheromatosis | 231 | 241 | 485 | 564 |
| Total no. angiography findings | 461 | 494 | 861 | 1078 |

*Distribution of angiography findings in women with right-sided breast cancer (BC), and left-sided BC. Radiotherapy (RT), left main coronary artery (LMCA), and number (no.).*

**Figure S-2**

**Risk of having a percutaneous coronary intervention stratified by pathological lymph node stage**

*Risk of having a percutaneous coronary intervention (PCI) in women with breast cancer (BC) receiving left-sided radiotherapy (RT) compared to right-sided RT stratified by pathological lymph node stage (N). Left anterior descending artery (LAD), right coronary artery (RCA), left main coronary artery (LMCA), left circumflex artery (LCx), hazard ratio (HR), confidence interval (CI), number (No.), and versus (vs).*

**Figure S-3**

**Risk of having a percutaneous coronary intervention stratified by type of surgery**

**

*Risk of having a percutaneous coronary intervention (PCI) in women receiving left-sided RT compared to right-sided RT stratified by type of surgery. Breast conserving surgery (BCS), pathological lymph node stage (N), left anterior descending artery (LAD), right coronary artery (RCA), left main coronary artery (LMCA), left circumflex artery (LCx), hazard ratio (HR), confidence interval (CI), versus (vs), and breast cancer (BC).*
